# Methamphetamine-induced changes in the striatal dopamine pathway in μ-opioid receptor knockout mice

**DOI:** 10.1186/1423-0127-18-83

**Published:** 2011-11-10

**Authors:** Sang Won Park, Xine Shen, Lu-Tai Tien, Richard Roman, Tangeng Ma

**Affiliations:** 1 Department of Pharmacology and Toxicology, University of Mississippi Medical Center, Jackson, MS 39216, USA; 2 Department of Physiology, Medical school, Soochow University, Jiangsu, P.R China; 3 School of Medicine, Fu Jen Catholic University, Taipei, Taiwan

**Keywords:** Amphetamine, μ-opioid receptor, addiction, dopamine receptors

## Abstract

**Background:**

Repeated exposure to methamphetamine (METH) can cause not only neurotoxicity but also addiction. Behavioral sensitization is widely used as an animal model for the study of drug addiction. We previously reported that the μ-opioid receptor knockout mice were resistant to METH-induced behavioral sensitization but the mechanism is unknown.

**Methods:**

The present study determined whether resistance of the μ-opioid receptor (μ-OR) knockout mice to behavioral sensitization is due to differential expression of the stimulatory G protein α subunit (Gαs) or regulators of G-protein signaling (RGS) coupled to the dopamine D1 receptor. Mice received daily intraperitoneal injections of saline or METH (10 mg/kg) for 7 consecutive days to induce sensitization. On day 11(following 4 abstinent days), mice were either given a test dose of METH (10 mg/kg) for behavioral testing or sacrificed for neurochemical assays without additional METH treatment.

**Results:**

METH challenge-induced stereotyped behaviors were significantly reduced in the μ-opioid receptor knockout mice when compared with those in wild-type mice. Neurochemical assays indicated that there is a decrease in dopamine D1 receptor ligand binding and an increase in the expression of RGS4 mRNA in the striatum of METH-treated μ-opioid receptor knockout mice but not of METH-treated wild-type mice. METH treatment had no effect on the expression of Gαs and RGS2 mRNA in the striatum of either strain of mice.

**Conclusions:**

These results indicate that down-regulation of the expression of the dopamine D1 receptor and up-regulation of RGS4 mRNA expression in the striatum may contribute to the reduced response to METH-induced stereotypy behavior in μ-opioid receptor knockout mice. Our results highlight the interactions of the μ-opioid receptor system to METH-induced behavioral responses by influencing the expression of RGS of dopamine D1 receptors.

## Background

Methamphetamine (METH) is a highly abused CNS stimulant with high reward properties that leads to compulsive drug seeking behavior [1,2]. The mechanism of the additive properties to METH remains to be determined. Repeated administration of METH results in behavioral sensitization characterized by persistent hyperlocomotor activity and stereotyped behaviors [3,4]. Animals remain sensitized for many weeks, suggesting that the development of sensitization involves long-lasting neuronal adaptations [5]. The neural alterations underlying behavioral sensitization are also thought to contribute to mimic changes associated with the compulsive drug seeking behavior. Thus, behavioral sensitization is widely used as an animal model for the study of drug addiction [5-8] and it is extremely important to find therapeutic agents for behavioral sensitization to psychostimulants.

The dopamine system is generally considered a main target for amphetamines to stimulate locomotor activity and stereotyped behaviors. The nigrostriatal dopaminergic pathway consists of dopaminergic neurons of the substantia nigra that innervate the striatum [9] that is intimately linked to the stereotyped behaviors produced by psychomotor stimulants [10]. It is well known that an increase in dopaminergic activity in the central nervous system (CNS) plays a central role in induction and expression of behavioral sensitization by psychomotor stimulants. For example it is known that activation of dopamine receptors is required for the expression of behavioral sensitization by METH [11]. METH stimulates the release of dopamine from dopaminergic neurons and activates dopamine receptors [12]. Dopamine receptors as members of the G protein-coupled receptor (GPCR) superfamily elicit a variety of cellular and behavioral responses through various signaling pathways to induce behavioral effects [13]. Regulators of G-protein signaling (RGS) proteins negatively regulate GPCR signaling, changes in RGS protein levels in the brain are thought to modulate the intensity and duration of signaling of cognate receptors [14]. The expression of several RGS proteins in the brain is rapidly altered in response to psychostimulants [15]. In addition there is growing evidence that exposure to amphetamine-like stimulants influences the expression of dopamine receptors, G-proteins and RGS in neurons that may contribute to stimulant-mediated behavioral responses [16]. Chronic administration of dopamine D1 agonist SKF 38393 results in enhanced behavioral responses to subsequent administration of a variety of dopamine agonists [17,18]. Others have found that stereotypic behavior in response to amphetamine administration is associated with increased expression of dopamine D1 receptors [19] and hypersensitivity of adenylate cyclase to dopamine stimulation which is blocked by the dopamine D1 antagonist SCH 23390 [20].

It is also clear that there are extensive anatomical and functional interactions between the dopaminergic system and other neuronal pathways. For example both the opioidergic and glutamatergic systems contribute to the development and maintenance of behavioral sensitization to METH [21]. Endogenous opioid systems have been found to play important roles in reward, positive reinforcement, and additive effects on drugs of abuse [22-24]. The endogenous opioid systems consist of a variety of endogenous opioid peptides and receptors. At least three opioid receptor subtypes (δ, μ, and κ) are currently recognized [25]. Enkephalins have high affinity for μ- and δ- opioid receptors whereas dynorphins have high affinity for κ-opioid receptors. It has been reported that amphetamines induce an increase in expression of the opioid peptide enkephalin precursor preproenkephalin mRNA in rodent striatum [26]. No behavioral sensitization to amphetamine was detected in the enkephalin knockout mice [27]. We also reported that μ-opioid receptor (μ-OR) knockout mice were less sensitive to the development of behavioral sensitization to METH [28]. However, it remains to be determined how μ-OR contribute to METH-induced behavioral responses. The present study examined whether METH exposure causes differential changes in the expression of stimulatory Gα (Gαs; subunit coupled to dopamine D1 receptors) or RGS associated with dopamine D1 receptors in the CNS that may contribute to the resistance to METH-induced behavioral sensitization in μ-OR knockout mice.

## Materials and methods

### Animals and drug treatments

The μ-OR knockout mice were originally developed by Loh *et al*. [29] on a C57/BL6 and 129/Ola hybrid genetic background. Our colony was maintained as heterozygotes by brother sister mating in the Laboratory Animal Facility of the University of Mississippi Medical Center (UMMC). All procedures were approved by Institutional Animal Care and Use Committee of the UMMC, and performed in compliance with the NIH Guide for the Care and Use of Laboratory Animals. Adult male wild-type and μ-OR knockout mice were used in this study. μ-OR knockout and wild-type mice (n = 12 for each genotype) were given METH (10 mg/kg, *i.p*.) once a day for 7 consecutive days to induce sensitization in order to investigate METH-evoked behavioral response. This dose was chosen on the basis of previous studies indicating that it was the dose that induced stereotyped behavioral sensitization to subchronic administration of METH in mice [28,30]. On day 11, after a 4 day drug washout period, the sensitized mice were challenged with a *i.p*. injection of METH (10 mg/kg). Stereotyped behaviors were monitored for 30 min before and for 5 hrs after the injection to evaluate the behavioral responses.

The behavior of mice was monitored in a Plexiglas^® ^box equipped with a CCD camera and recorded on video tape, which was subsequently analyzed by a trained observer. The intensity of stereotyped activity was scored on 4-point scale (0 - normal behavior, 1 - periodic sniffing, 2 - continuous sniffing, 3 - continuous sniffing, periodic licking or gnawing, 4 - continuous licking or gnawing) as described by Costall and colleagues [31].

Parallel experiments were performed in another group of wild-type and μ-OR knockout mice to assess changes in the expression of dopamine receptors and mRNA in the brain. These animals were sensitized using the same 7 day exposure to METH or vehicle. After a 4 day drug-washout period, the animals were decapitated and the brains collected and frozen in liquid nitrogen. Coronal sections (14-20 mm thick) were cut using a cryostat (Tissue-Tek, cyro 2000) at -20°C, thaw-mounted on gelatin-coated slides and stored at -80°C for autoradiography and *in situ *hybridization analysis.

### Autoradiography

Dopamine D1 receptor levels were measured using radiolabeled ligand binding and autoradiography as previously described by Qian *et al*. [32]. Briefly, brain sections were pre-incubated at 4°C for 30 min in a 50 mM Tris-HCI buffer (pH 7.4) containing 120 mM NaCl, 5 mM KCl, 2 mM CaCl_2_, and 1 mM MgCl_2_, and then incubated for 60 min with 1.6 nM of the labeled dopamine D1 receptor antagonist [^3^H]SCH23390 at room temperature. Other sections were incubated with 30 μM of the dopamine D1 receptor ligand (±)SKF38393 [33] to control for nonspecific binding. The labeled brain sections as well as a set of [^3^H]-impregnated plastic standards ([^3^H]Microscale, Amersham Life Science) were placed on Kodak BioMax MS film for 3 weeks (-80°C). The films were developed, and then analyzed using a scanning densitometer and Image Quant 3.3 program (Molecular Dynamics; Sunnyvale, CA).

### *In Situ *Hybridization

The expression of Gαs, RGS2, and RGS4 mRNAs in the brain were determined using *in situ *hybridization. The focus of the present experiment was to examine changes in the expression of Gαs, RGS2, and RGS4 in the striatum of the mouse brain. We and others have used in situ hybridization techniques [34-36] to successfully study gene expression for a wide variety of gene products. The technique relies on the specificity of the probe. Oligonucleotide probes complementary to mRNAs encoding mouse Gαs (5'-GCAAAGCAGCGCCTGCCTGCCCGTCTGCCTGCCGCCGCC-3')[34], RGS2 (5'-GGGCTCCGTGGTGATCTGTGGCTTTTTACATAAG-3'), and RGS4 (5'-GCTGGAAGGATTGGTCAGGTCAAGATAGAATCGAG-3')[35] were 3' end labeled with [^35^S]dATP using terminal deoxynucleotidyltransferase (PerkinElmer Life Sciences, Shelton, CT) and *in situ *hybridization was performed as described earlier [36]. The probes used were identical to those described by Tervonen *et al*. [35] who verified that they specifically bound to RGS2 and RGS4 and Przewlocka *et al*. [34] who tested the Gαs probe. We also BLASTED the sequence of the probes against all of the sequences in GENBANK and found that they exhibited a 100% match to the intended target. Only the RGS4 probe exhibited any significant homology (17 of 35 bp) to another target, i.e. the presenilin-2 gene. However, given the limited numbers of complementary base pairs, it is highly unlikely that the RGS4 would bind to this target at the hybridization temperature used of 38°C. Moreover, we also performed appropriate control experiment to exclude non-specific binding. The labeled slides were exposed to Kodak BioMax MR films for 5 days for Gαs or 11 weeks for RGS2 and RGS4, and the films were developed and fixed. The quantification of the autoradiogram was performed using the Image Quant software (Molecular Dynamics, Sunnyvale, CA).

### Statistical Analysis

Data are expressed as mean values ± SEM. The significance of differences in mean values was analyzed using a *t *test (stereotyped behaviors) or a two-way ANOVA followed by a Student-Newman-Keuls post hoc test. A *P *< 0.05 was considered to be significant.

## Results

### METH-evoked stereotyped behaviors in METH-sensitized wild-type and μ-OR knockout mice

**Figure 1:**
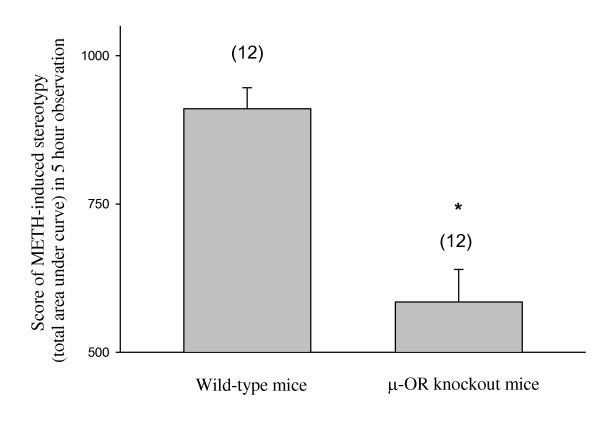
**METH (10 mg/kg)-evoked stereotyped behaviors in wild-type and μ-OR knockout mice that were exposed to METH for 7 days**. METH (10 mg/kg)-evoked stereotyped behaviors in wild-type and μ-OR knockout mice that were exposed to METH for 7 days. Mean values ± SEM are presented. Numbers in parentheses represent the number of animals studied. * indicates a significant difference (*P *< 0.05) from the corresponding value in METH-sensitized wild-type mice.

Administration of METH in sensitized wild-type animals produced stereotyped behaviors, characterized by continuous sniffing and licking that persisted for about 5 hours. In the μ-OR knockout mice the cumulative score of stereotyped behaviors was significantly lower than in the wild-type mice (Figure 1).

### [^3^H]SCH23390 binding in the striatum of METH-sensitized wild-type and μ-OR knockout mice

**Figure 2:**
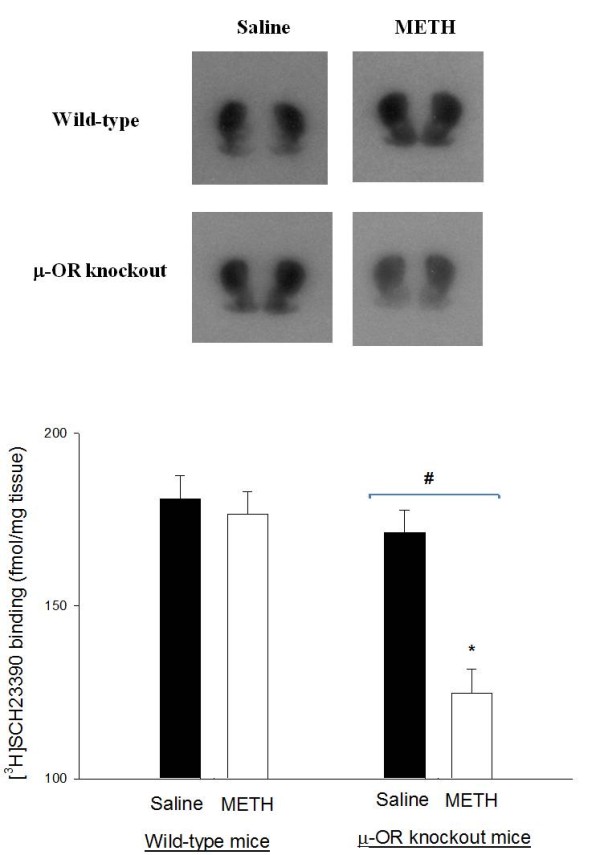
**Binding of dopamine D1 ligand [^3^H]SCH23390 in the brains of METH-sensitized wild-type and μ-OR knockout mice**. Binding of dopamine D1 ligand [^3^H]SCH23390 in the brains of METH-sensitized wild-type and μ-OR knockout mice. Both strains of mice were pretreated with daily injections saline or METH (10 mg/kg) for 7 consecutive days. Mice were killed 4 days after the final injection and brain tissues were taken for autoradiographic analysis of [^3^H]SCH23390 binding. Representative autoradiograms of [^3^H]SCH23390 binding are shown on the top. Mean values ± SEM are presented. Numbers in parentheses represent the number of animals/brains studied. * indicates a significant difference (*P *< 0.05) from METH-sensitized wild-type mice; # indicates a significant difference (*P *< 0.05) from saline-treated μ-OR knockout mice.

Representative autoradiograms of [^3^H]SCH23390 binding in the brain of wild-type and μ-OR knockout mice are presented in Figure 2. High levels of [^3^H]SCH23390 binding were seen in the striatum. Basal binding of [^3^H]SCH23390 in the striatum was not significantly different between wild-type and μ-OR knockout mice treated with saline. Repeated METH treatment had no significant effect on D1 receptor binding in wild type mice. In contrast, the binding of [^3^H]SCH23390 was markedly reduced in the μ-OR knockout in mice sensitized by repeated exposure to METH.

### The expression of the stimulatory G protein α subunit (Gαs) mRNA in the striatum of METH-sensitized wild-type and μ-OR knockout mice

**Figure 3:**
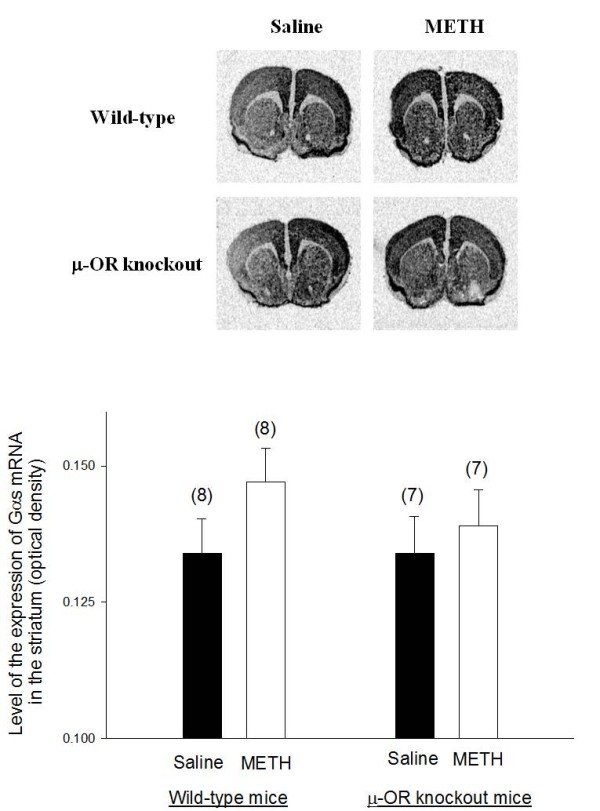
**The expression of Gαs mRNA in the brains of METH-sensitized wild-type and μ-OR knockout mice**. The expression of Gαs mRNA in the brains of METH-sensitized wild-type and μ-OR knockout mice. Animal treatments were the same as described in Fig. 2. Gαs mRNA levels in the brain sections were analyzed by *in situ *hybridization analysis. Representative autoradiograms of Gαs mRNA expression in the brain of mice are presented at the top of the figure. Mean values ± SEM are presented. Numbers in parentheses represent the number of animals/brains studied.

Representative autoradiograms of *in situ *hybridization of Gαs mRNA in the brain of wild-type and μ-OR knockout mice are presented in Figure 3. Gαs mRNA is widely expressed in most brain areas including striatum and cerebral cortex. The expression of Gαs in the striatum was similar in both wild-type and μ-OR knockout animals treated with saline. METH treatment did not alter the expression of Gαs mRNA in the striatum wild type mice or in μ-OR knockout mice.

### The expression of RGS2 and RGS4 mRNAs in the striatum of METH-sensitized wild-type and μ-OR knockout mice

**Figure 4:**
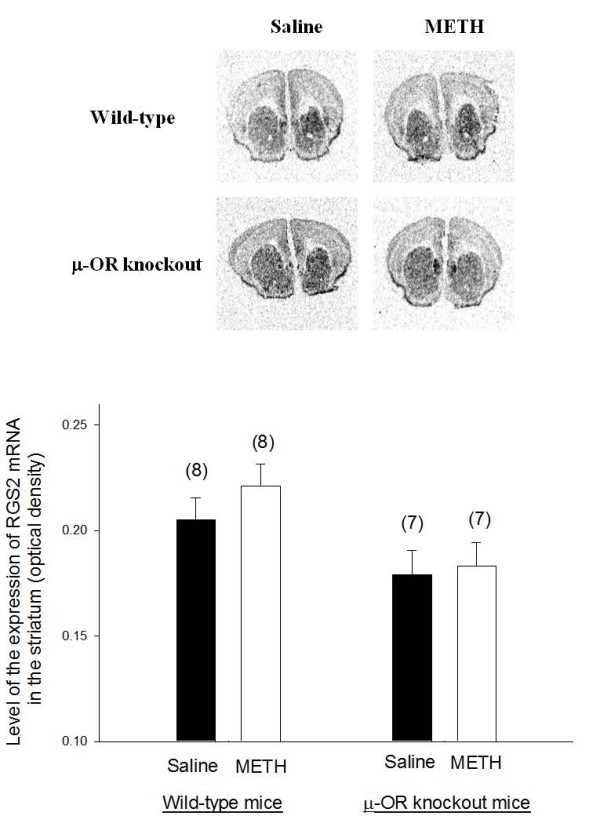
**The expression of RGS2 mRNA in the brains of METH-sensitized wild-type and μ-OR knockout mice**. The expression of RGS2 mRNA in the brains of METH-sensitized wild-type and μ-OR knockout mice. Animal treatments and preparation of brain sections for analysis of RGS2 mRNA levels were the same as described in Fig. 3. Representative autoradiograms of RGS2 mRNA expression in the brain of mice are presented at the top of the figure. Mean values ± SEM are presented. Numbers in parentheses represent the number of animals/brains studied.

**Figure 5:**
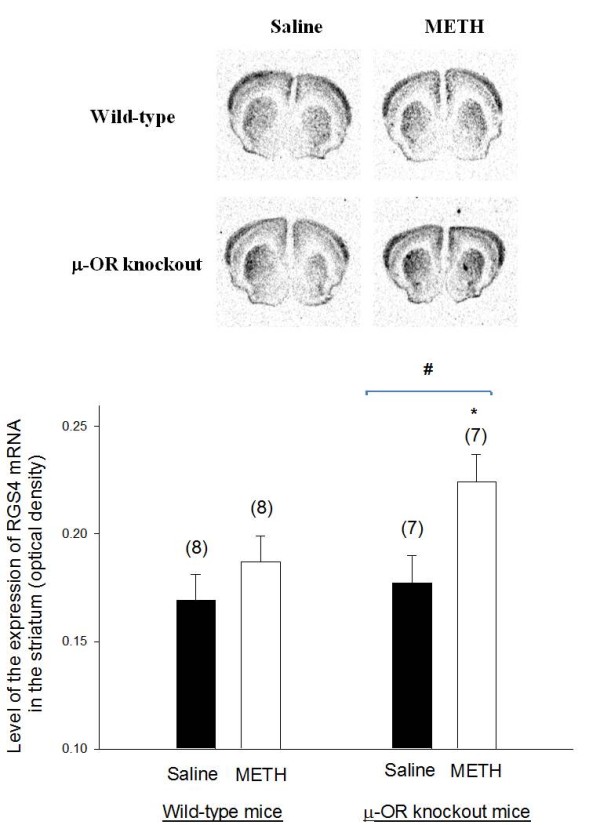
**The expression of RGS4 mRNA in the brains of METH-sensitized wild-type and μ-OR knockout mice**. The expression of RGS4 mRNA in the brains of METH-sensitized wild-type and μ-OR knockout mice. Animal treatments and preparation of brain sections for analysis of RGS4 mRNA levels were the same as described in Fig. 3. Representative autoradiograms of RGS4 mRNA expression are shown at the top. Mean values ± SEM are presented. Numbers in parentheses represent the number of animals/brains studied. * indicates a significant difference (*P *< 0.05) from METH-sensitized wild-type mice; # indicates a significant difference (*P *< 0.05) from saline-treated μ-OR knockout mice.

Representative autoradiograms of *in situ *hybridization signals for RGS2 and RGS4 mRNAs in the brain of wild-type and μ-OR knockout animals are presented in Figures 4 and 5, respectively. Both of RGS2 and RGS4 mRNAs were highly expressed in the striatum. There was no significant difference in the expression of RGS2 mRNA in the striatum of μ-OR knockout or wild-type mice treated with saline or METH. Basal expression of RGS4 was also similar in μ-OR knockout and wild-type mice treated with saline. However, the expression of RGS4 mRNA in the striatum increased in μ-OR knockout mice treated with METH but remained unchanged in wild-type mice sensitized with METH.

## Discussion

The CNS stimulant-METH induces behavioral sensitization which is associated persistent hyperlocomotor activity and stereotyped behaviors. Behavior sensitization is a widely used in rodents model for study of drug addiction and drug seeking behaviors [5,37]. In the present study we confirmed previous finding that μ-OR knockout mice demonstrate significantly decreased behavioral sensitization to METH as compared with wild-type mice. This was associated with a significant reduction in dopamine D1 receptor density in the striatum of approximately 30% in μ-OR knockout mice when compared to wild-type mice exposed to METH. By way of contrast, METH had no effect on dopamine D1 receptor density in the striatum of wild-type mice.

We also found that the expression of Gαs mRNA was unaltered by METH exposure in wild type or knockout mice, as was the expression of mRNA of the regulator of G-protein signaling, RGS2. However, the expression of RGS4 mRNA was significantly increased in the striatum of METH treated μ-OR knockout mice as compared to saline treated controls, whereas METH treatment had no effect on RGS4 mRNA in wild-type controls. These data suggest that in μ-OR knockout mice dopamine D1 receptor function in the striatum can be more readily down-regulated than in wild- type mice after repeated METH exposure. This may, in part, explain the decreased behavioral sensitization observed after METH treatment of μ-OR knockout mice.

Dopamine is an important neurotransmitter in the CNS where it plays essential roles in numerous physiological, neuronal, and behavioral processes. One important component of the pathways in the CNS is the nigrostriatal dopaminergic system, projecting from the substantia nigra to the striatum (putamen and caudate nucleus) that is known to be crucial for the induction of stereotyped behaviors [10]. Previously, we and others have performed dose response studies and found that 2.5 mg/kg METH is sufficient to elicit a locomotor response [28,38] but higher doses (10 mg/kg) are needed to induce behavioral sensitization to METH [28,30]. Repeated stimulation of dopamine receptors with agonists has been shown to cause down-regulation in expression of these receptors [39]. As an indirect dopamine receptor agonist, METH is known to stimulate the release and inhibit reuptake of dopamine from synaptic cleft [40], increasing extracellular dopamine levels and activating postsynaptic striatal dopamine receptors. Thus, repeated METH exposure lead to down regulation of the expression of dopamine receptors in the striatum. In other studies, we found that METH (10 mg/kg) was associated with decreased tyrosine hydroxylase (the rate limiting enzyme of dopamine synthesis) in wild-type mice but not in the μ-OR knockout mice. These results along with our present findings indicate that the changes of dopaminergic system in mice chronically exposed to METH is related to a decrease in the expression of the enzyme involved in the synthesis of dopamine and its actions on dopamine D1 receptors rather than to the loss of dopaminergic neurons. Nonetheless, these data demonstrate that the μ-opioid receptor modulates the response of dopamine neurons to METH.

There are two types of dopamine receptors in the striatum: D1 and D2. Striatonigral neurons largely express dopamine D1 receptors whereas most striatopallidal neurons express dopamine D2 receptors [41]. Although concurrent activation of dopamine D1 and D2 receptors is thought to be required for the full induction of stereotyped behaviors [11], activation of dopamine D1 receptors is primarily responsible for the induction of dopamine-mediated stereotypy [42]. Therefore, the down regulation of dopamine D1 receptor we found in the striatum of METH-sensitized μ-OR knockout mice compared with wild-type is consistent with the view that this contributes to the less of METH-induced stereotyped behaviors in this strain of mice. Surprisingly, METH exposure in wild-type mice did not down-regulate D1 dopamine receptors. Previously our lab reported that quantitative autoradiographic analysis of striatum and nucleus accumbens showed that METH treatment leads to a decrease in dopamine D1 receptor ligand binding in μ-OR knockout mice but not in wild-type mice at low concentration (0.4 nM) of dopamine D1 receptor antagonist SCH 23390 [36]. This suggests that interactions between opiodergic receptors/neurons and neurons of the nigrostriatal pathway occur that stabilize receptor density. These interactions between these pathways and the mechanisms involved deserve further study.

Chronic treatment (2-3 weeks) with dopamine D1 receptor antagonist SCH 23390 has been reported to increase the expression of mRNA for preproenkephalin in the rat striatum [43,44]. Recently, we found that there was an increase in expression of preproenkephalin mRNA in the nucleus accumbens and striatum in METH-sensitized wild-type mice but not in μ-OR knockout mice [36]. Also, METH induced hyperlocomotor activity at low doses and stereotyped behaviors at high doses in wild-type mice [28] but not in μ-OR knockout mice. These results indicate that a decrease in striatal and nucleus accumbens D1 receptors in METH-sensitized μ-OR knockout mice is associated with a decrease in the behavioral response in these animals. The exact mechanism of how the μ-opioid system modulates dopaminergic neurotransmission and thus influences METH-produced behavioral responses is unclear. Based on data in the literature, however, it can be proposed that METH-induced changes in G protein signaling and RGS proteins might play a role in the development of behavioral sensitization to the drug.

Dopamine receptors are members of the GPCR family. Stimulation of the dopamine D1 receptors activates adenylyl cyclase via Gαs, increasing intracellular cAMP that activates cAMP-dependent protein kinase A and its down-stream effectors [45]. There is evidence that G protein signaling may be disrupted in drug addiction and neuropsychiatric disorders [46,47]. For example, postmortem brain studies have revealed increased levels of Gαs in bipolar disorder, a type of mood disorder with unknown etiology as well as being inducible by CNS stimulants [48]. Elevation of Gαs levels is thought to enhance signaling through the dopamine D1 receptor and contribute to dopamine D1 receptor activation-mediated behavioral responses [49]. Therefore, we examined the expression of Gαs mRNA in the striatum of METH-sensitized mice. The results of the present study indicate that the expression of Gαs mRNA in the striatum was not altered by repeated METH exposure in either μ-OR knockout or wild-type mice.

Another possible effect of repeated METH exposure is to alter the activity of the Gα protein. The primary regulators of GTPase activity of Gα-subunits are RGS proteins that rapidly terminate receptor-activated GαGTP signaling by accelerating the hydrolysis of GTP to GDP [50,51]. More than 30 mammalian RGS proteins have been identified [50,52]. Gene expression studies demonstrate that RGS2 and RGS4 are avidly expressed in cortex, striatum, and several thalamic regions of the brain [53,54]. The available evidence suggests that activation of dopamine D1 and D2 receptors in the striatum of rat is coupled to RGS2 and RGS4 [55].

Amphetamine-like stimulants alters RGS mRNA expression in the brain and triggers GPCR signaling [56-58]. There are several lines of evidence that acute or repeated treatment with amphetamine modulates drug-induced behavioral and changes in gene and protein expression of RGS4 in prefrontal cortex and dorsal striatum [59-61]. RGS4 mRNA was decreased in the striatum lasting from 1 to 6 hr after acute amphetamine [62]. RGS4 may belong to the growing family of factors regulating convergence of dopamine signaling in the striatum [63].

In the present study, METH (10 mg/kg) exposure had no influence on the expression of RGS2 mRNA in the striatum of either μ-OR knockout or wild-type mice. However, there was a higher expression of RGS4 mRNA in the striatum of METH-sensitized μ-OR knockout mice but not of wild-type mice. Increased expression of RGS4 is consistent with a reduction in signaling via dopamine D1 receptors in the striatum that may already be reduced due to the decreased density of dopamine D1 receptors in METH treated μ-OR knockout mice. Down-regulation of dopamine D1 receptor binding in combination with increased RGS4 mRNA levels is consistent with diminished dopamine D1 receptor function in METH-exposed μ-OR knockout mice that would decrease the occurrence of behavioral sensitization.

## Conclusions

In conclusion, the present study indicates that knockout of μ-OR in mice reduces their sensitivity to METH-induced stereotyped behaviors. Down-regulation of the expression of the dopamine D1 receptor in combination with up-regulation of the expression of RGS4 in the striatum of METH-sensitized μ-OR knockout mice may contributes to the resistance to the behavioral responses to METH in this strain. The results suggest that the μ-opioid system and RGS proteins might be targets for the development of drugs that might reduce the reward potential and compulsive drug seeking behavior in METH abusers.

## Competing interests

The authors declare that they have no competing interests.

## Authors' contributions

SP carried out the *in situ *hybridization studies, performed the statistical analysis, and drafted the manuscript. XS participated in the animal treatment and study. LT carried out the ligand binding assay. RR participated in the data analysis and editing the manuscript. TM conceived the study and supervised the study. All authors read and approved the final manuscript.
